# Macular pucker, an atypical clinical presentation of ocular toxoplasmosis: a case report

**DOI:** 10.1186/s12886-021-01983-7

**Published:** 2021-05-17

**Authors:** Si Zhang, Chun-yan Xue, Ya-jun Liu, Wen-wen Zhang, Zheng-gao Xie

**Affiliations:** 1 Department of Ophthalmology, Nanjing Drum Tower Hospital, The Affiliated Hospital of Nanjing University Medical School, Nanjing, 210008 Jiangsu Province China; 2 Department of Ophthalmology, Jinling Hospital, Nanjing University School of Medicine, Nanjing, 210008 Jiangsu Province China

**Keywords:** Epiretinal membrane, Macular pucker, Ocular toxoplasmosis, Toxoplasma gondii, Intraocular fluid testing, Serologic testing

## Abstract

**Background:**

Ocular toxoplasmosis caused by Toxoplasma gondii is an infectious disease which is widely distributed around the world and can present with various clinic manifestations. We are here reporting an unusual case presented with epiretinal membrane (ERM), i.e., macular pucker.

**Case presentation:**

A 16-year old male patient visited our outpatient clinic complaining of decreased vision for about 8 years in his left eye. The best-corrected visual acuity (BCVA) was 20/20 OD and 20/400 OS. There was sensory exotropia in his left eye. No inflammatory cells or flare were found in his anterior chamber or vitreous cavity OU. An ERM involving his left macular area was found on his dilated fundus exam, which was confirmed by Optical Coherence Tomography (OCT). The ERM was found to involve his left macular area with his foveal ellipsoid zone absent. The right eye was found to be within normal limit.

After a thorough discussion with the patient and his parents about treatment options and surgical benefits, risks and alternatives, we performed vitrectomy, peeled off the ERM and collected the vitreous sample for parasite testing during the procedure. Patient’s blood also was drawn for serological testing. Vitreous sample analysis and serological tests confirmed ocular toxoplasmosis OS as his final diagnosis. Unfortunately, the BCVA of this patient was not improved after the surgery, but the exotropia disappeared.

**Conclusion:**

ERM is an unusual clinical presentation of ocular toxoplasmosis. We may add Toxoplasma gondii infection as a differential diagnosis when encountering ERM cases.

## Background

Toxoplasmosis is an infectious disease caused by the obligate intracellular parasite Toxoplasma gondii, which is widely distributed around the world. About 30% of people in the world are infected with toxoplasmosis [1]. Cats are the only definitive host, while human and other animals are the intermediate hosts. The transmission routes of Toxoplasma include ingestion of food and water contaminated by cysts, blood transfusion, organ transplantations and transplacental transmission [2]. Patients with active disease frequently present with retinochoroidal lesions. Active toxoplasmic retinochoroiditis most often involves the posterior pole (76%), and is more common found in the unilateral eyes. More than 90% of patients have normal visual acuity in the contralateral eye [3]. Ocular toxoplasmosis can cause various complications, including exudative retinal detachment, retinal vascular occlusion, choroidal neovascularization, pre-retinal membrane, macular hole and macular edema [4–7]. Our case showed an atypical manifestation, a white oval-shaped ERM in the involved eye. We reviewed all cases of ocular toxoplasmosis in the literature, secondary ERM as the main presentation has not yet been reported. We are reporting this rare case as reminding for considering the possibility of Toxoplasma gondii infection for ERM in adolescent patients.

## Case presentation

**Fig. 1:**
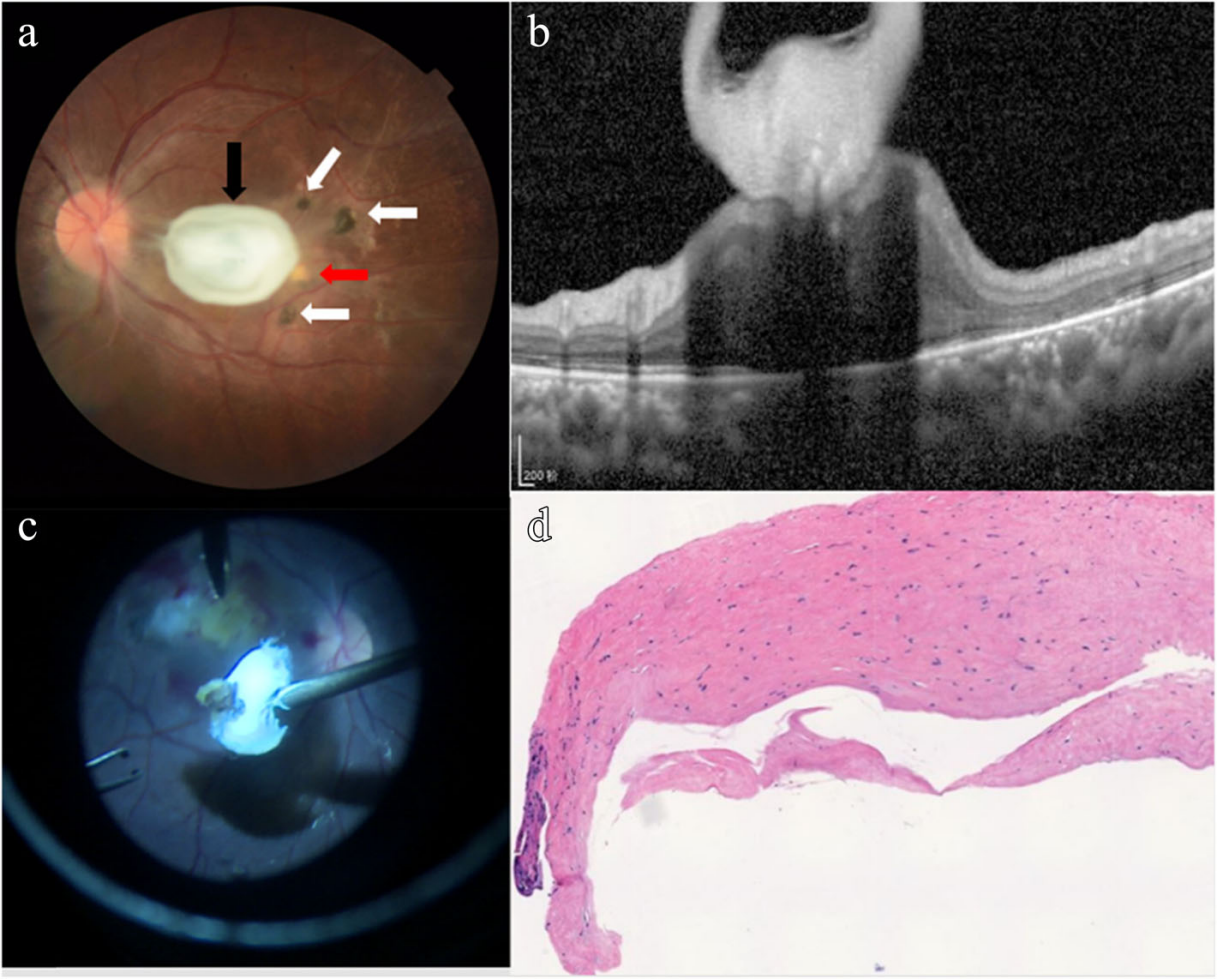
Fundus photograph. A white oval-shaped thickened ERM (black arrow), pigmented chorioretinal scars (white arrows) and yellow-white round retinochoroidal lesions (red arrow) temporal to macula (**a**). Spectral-domain optical coherence tomography (SD-OCT). ERM and traction involving the macula, ellipsoid zone defects in the central macula (**b**). Intraoperative findings. ERM was peeled off, a brown pigmented lesion attached beneath (**c**). Histopathological examination of the ERM, hyalinosis of local organization found in the samples (HE× 100) (**d**)

A 16-year old male patient visited our outpatient clinic with a chief complaint of decreased vision for about 8 years in his left eye. He had a history of close contact with cats and dogs, with no history of any other systemic illness, trauma or surgery. The best-corrected visual acuity (BCVA) was 20/20 in his right eye and 20/400 in his left eye. The intraocular pressure was normal in both eyes. There was sensory exotropia in his left eye. No inflammatory cells were found in his anterior chamber or vitreous OU. The fundus exam of his left eye showed a white oval-shaped ERM with the size of of 2.0 papillary diameter, some pigmented chorioretinal scars, and yellow-white round retinochoroidal lesions on the temporal side of the macula (Fig. 1a). Spectral-domain optical coherence tomography (SD-OCT) confirmed a thickened ERM with vitreous retinal traction involving the macula, and ellipsoid zone defects in the central macula (Fig. 1b).

A preliminary diagnosis of parasitic infection was made based on the choroidoretinopathy in the posterior pole of his left eye. To further confirm this diagnosis, 2.0 ml of vitreous humor was collected during the following vitrectomy and sent for parasite testing. Meanwhile, his blood sample was also collected and sent for serological testing. During the surgery, the ERM was peeled off with caution, together with a brown pigmented lesion attached beneath (Fig. 1c). The proliferative membranes were sent for histopathological examination, which later on reported “hyalinosis of local organization found in the samples” (Fig. 1d). Vitreous sample analysis found Toxoplasma DNA undetectable, but Toxoplasma IgG to be 6.95 IU/ml (reference range less than 4 IU/ml). His serum Toxoplasma IgG was found to be 20 IU/ml (reference range was less than 20 IU/ml). The Goldmann-Witmer coefficient (GWC) value of Toxoplasma gondii was 36.72 (reference range 0–2). Based on those test results listed above, the final diagnose was ocular toxoplasmosis involving his left eye.

**Fig. 2:**
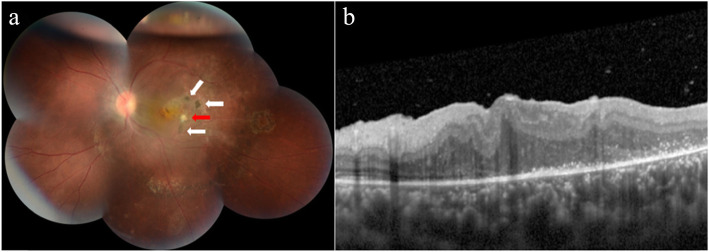
Fundus photography after surgery and SD-OCT scan of the patient. The pigmented chorioretinal scars (white arrows) and yellow-white retinochoroidal lesions (red arrow) at temporal macula had no obvious changes (**a**). Reduction in central macular thickness (**b**)

Unfortunately, 1 month after the surgery, the patient’s BCVA was still 20/400 (no effect of correction). His intraocular pressure was still within normal limits. Unexpectedly, his left exotropia resolved after the surgery. The lens and vitreous remained unremarkable. Postoperative fundus examination (Fig. 2a) revealed no obvious changes of the pigmented chorioretinal scars and yellow-white retinochoroidal lesions on the temporal side of the macula. A repeat SD-OCT showed a reduction in central macular thickness (Fig. 2b).

## Discussions and conclusions

In this case, the patient came from a rural area, with a history of cats and dogs close contact, and a habit of drinking cold unboiled tap water and eating raw vegetables since his childhood. These all put him at risk for toxoplasmosis infection. Several pigmented chorioretinal scars and yellow-white retinochoroidal lesions were found in the posterior pole of the left eye, suggested the possibility of ocular toxoplasmosis, even though there were no inflammatory cells found in the anterior chamber and vitreous cavity. The preliminary diagnosis was later on confirmed by further serologic and vitreous analysis.

Toxoplasma gondii transmission can be congenital (transplacental) or postnatally acquired [8]. Congenital infection refers to the transmission of Toxoplasma to the fetus through the placenta by pregnant women suffering from acute toxoplasmosis or reinfection with toxoplasmosis. Children are mostly asymptomatic at birth and may later on develop visual loss, nystagmus, sensory exotropia, and neurological symptoms, such as cerebral edema, brain calcification, and/or intellectual disability. Our patient reported here had normal visual acuity in both eyes and nervous system development since childhood reported by his parents and presented with a loss of vision at the age of eight, so congenital Toxoplasma gondii infection could be excluded. Acquired Toxoplasma gondii infection in children is often difficult to diagnose, because of the absence of accurate and timely complaints due to patients’ young age. Therefore, there is great difficulty in early diagnosis and treatment of uveitis. If early diagnosis and treatment are not carried out, it may cause severe visual impairment [9]. In this case, the visual prognosis for the patient was poor due to macular destruction, possible amblyopia and sensory exotropia. Ocular toxoplasmosis is the most common type of pediatric posterior uveitis, accounting for up to 50% of posterior uveitis cases [10]. ERM secondary to uveitis is common, but ERM secondary to ocular toxoplasmosis as the main presentation has not been reported. The formation of the ERM is due to the inflammatory response that causes retinal pigment epithelium (RPE) cells, glial cells, Müller cells, fibroblasts to migrate and proliferate and secrete extracellular matrix [11]. Miranda [12] reported 14 patients with macular ERM secondary to ocular toxoplasmosis, all of whom had a relatively transparent and thin type of ERM which were completely different from the white dense thick ERM found in our case. All patients underwent vitrectomy combined with ERM peeling. The average time from diagnosis to surgery was 36.57 ± 24.40 months. The results showed that 12 patients presented with different degrees of improved visual acuity, and 2 patients had no improvement in visual acuity. Adan [13] reported 2 patients with ERM secondary to ocular toxoplasmosis who underwent vitrectomy combined with ERM peeling. Postoperative SD-OCT showed that the macular foveal traction was relieved, and both patients had visual acuity improvement without postoperative complications.

In our case, the ERM covering the macular area of the patient’s left eye was accompanied by severe visual loss with sensory exotropia. After thorough preoperative examinations and detailed discussions of benefits, risks and alternatives with the patient and his parents, a middle and posterior vitrectomy combined with ERM peeling surgery was performed. One month after the surgery, the patient’s BCVA was still 20/400, but the exotropia vanished and the eye position was notably improved.

Currently, intraocular fluid and serologic testing of Toxoplasma gondii DNA, IgG and IgM are commonly used to diagnose Toxoplasma gondii infection. The patient’s vitreous humor and blood specimens were tested for Toxoplasma DNA, IgG and IgM. The results of intraocular fluid showed that Toxoplasma DNA was zero, Toxoplasma IgG was 6.95 IU/ml (reference range less than 4 IU/ml). His serum Toxoplasma IgG was 20 IU/ml (reference range was less than 20 IU/ml), and the GWC value of Toxoplasma was 36.72 (reference range 0 ~ 2). Because Toxoplasma gondii is an obligate intracellular parasite, the positive rate of Toxoplasma DNA detection in intraocular fluid is usually low, about 30–40% [14]. Although Toxoplasma DNA in the intraocular fluid of this patient was zero, Toxoplasma gondii infection was diagnosed when the GWC value was found by over four.

The classical treatment of ocular toxoplasmosis is triple therapy with pyrimethamine, sulfadiazine and prednisone [2]. For immunocompetent patients, toxoplasmic chorioretinitis is a self-limiting inflammation that resolves spontaneously within 4–8 weeks. Since no drug has been found to cure the infection in human hosts, the goal of antimicrobial treatment continues to be limiting parasite multiplication during active retinitis [15]. In this case, the patient with inactive ocular toxoplasmosis received no antimicrobial treatment after surgery considering the side effects of antiparasitic drugs. The patient was followed up 4 months after the surgery, his left eye was still in stable condition with the absence of active inflammation.

ERM, especially thick ERM, secondary to ocular toxoplasmosis as the main presentation is rare. Intraocular fluid and serologic testing can provide sufficient evidence for the diagnosis of toxoplasmosis. Vitrectomy combined with ERM peeling is an effective treatment for ERM secondary to ocular toxoplasmosis. This case serves as a reminder that the possibility of Toxoplasma gondii infection should be taken into consideration in pediatric ERM patients.

## Data Availability

All data supporting the conclusions of this article are included in the present article.

## Abbreviations

ERM: Epiretinal membrane
BCVA: Best-corrected visual acuity
SD-OCT: Spectral-domain optical coherence tomography
RPE: Retinal pigment epithelium
GWC: Goldmann-Witmer coefficient

## References

[1] Garza-Leon M, Garcia LA (2012). Ocular toxoplasmosis: clinical characteristics in pediatric patients. Ocul Immunol Inflamm.

[2] Montoya JG, Liesenfeld O (2004). Toxoplasmosis. Lancet.

[3] Chan NS, Choi J, Cheung CMG (2018). Pediatric uveitis. Asia Pac J Ophthalmol.

[4] Holland GN (2004). Ocular toxoplasmosis: a global reassessment. Part II: disease manifestations and management. Am J Ophthalmol.

[5] Rodríguez Á, Gómez FE, Valencia M (2015). Peripheral retinal neovascularization in recurrent cicatricial toxoplasmic retinochoroiditis: case series report. Eur J Ophthalmol.

[6] Arana B, Fonollosa A, Artaraz J (2014). Macular hole secondary to toxoplasmic retinochoroiditis. Int Ophthalmol.

[7] Papadopoulou DN, Petropoulos IK, Mangioris G (2011). Pars plana vitrectomy in the treatment of severe complicated toxoplasmic retinochoroiditis. Eur J Ophthalmol.

[8] Garza-Leon M, Muccioli C, Arellanes-Garcia L (2008). Toxoplasmosis in pediatric patients. Int Ophthalmol Clin.

[9] Lonngi M, Aguilar MC, Ríos HA (2016). Pediatric uveitis: experience in Colombia. Ocul Immunol Inflamm.

[10] Patel H, Goldstein D, FRCSC (2003). Pediatric uveitis. Pediatr Clin N Am.

[11] Raval V, Rao S, Das T (2018). Anatomical and functional outcomes of pars plana vitrectomy for inflammatory epiretinal membrane surgery in healed toxoplasmosis infection. Indian J Ophthalmol.

[12] Miranda AF (2016). Costa de Andrade G, Novais EA, et al. outcomes after pars plana vitrectomy for epiretinal membranes associated with toxoplasmosis. Retina..

[13] Adan A, Giralt J, Alvarez G (2009). Pars plana vitrectomy forvitreoretinal complications of ocular toxoplasmosis. Eur J Ophthalmol.

[14] Santos FF, Nascimento H, Muccioli C (2015). Detection of toxoplasma gondii DNA in peripheral blood and aqueous humor of patients with Toxoplasmic active focal necrotizing retinochoroiditis using real-time PCR. Arq Bras Oftalmol.

[15] Butler NJ, Furtado JM, Winthrop KL, Smith JR (2013). Ocular toxoplasmosis II: clinical features, pathology and management. Clin Exp Ophthalmol.

